# The challenges of Chile to achieve control the HIV/AIDS pandemic the year 2030: A review

**DOI:** 10.1097/MD.0000000000038288

**Published:** 2024-07-26

**Authors:** Pablo Ferrer Campos

**Affiliations:** a Molecular Medicine Laboratory, Hospital Clínico Universidad de Chile, Independencia, Santiago, Chile.

**Keywords:** HIV/AIDS, Chile HIV infection, 95/95/95 UNAIDS strategy

## Abstract

Chile is contending with the highest rates of new human immunodeficiency virus (HIV) cases in both Latin America and globally, despite substantial ongoing investments in treatment. This comprehensive study, derived from PUBMED and Google searches, ANID data, and various organizational reports, highlights key areas for improvement. Over the past decade, Chile’s annual infection rate has risen, signaling an urgent need for detailed analysis and effective solutions. The study includes 44 references, comprising 32 scientific articles and 12 reports from entities like the WHO and the Pan American Health Organization. Data was meticulously collected through diverse means, such as scientific congresses, meetings with authorities, and direct data requests. Fourteen critical points are identified for addressing the HIV epidemic in Chile, spanning from legislative reforms to enhanced prevention campaigns. Key recommendations include universal diagnosis, decentralized healthcare, the availability of self-tests, and a focus on mental health and the impact of migration. Despite Chile’s strong economic indicators, factors such as inadequate sexual education, outdated legislation, and centralized diagnostic processes contribute to the persistent increase in new cases. The study underscores the pressing need for enhanced investment in prevention policies. Chile faces significant challenges in meeting the 90/90/90 targets, yet there is optimism in aiming for the 95/95/95 strategy by 2030. Achieving success requires a global commitment, an emphasis on prevention, and collaborative efforts among authorities, healthcare providers, and patients. Overcoming these identified barriers is essential for Chile to reach its ambitious goal and ultimately end the HIV epidemic.

## 1. Introduction

Chile has the highest rate of new human immunodeficiency virus (HIV) cases in Latin America and worldwide and is one of the 10 countries showing an increase of more than 50% in new cases in the last 10 years. The Ministry of Health (MINSAL) reported that approximately 84,000 people are living with HIV in Chile in 2021, with an HIV prevalence of 0.5% among people aged 15 to 49 years.^[1,2]^

**Figure 1.**
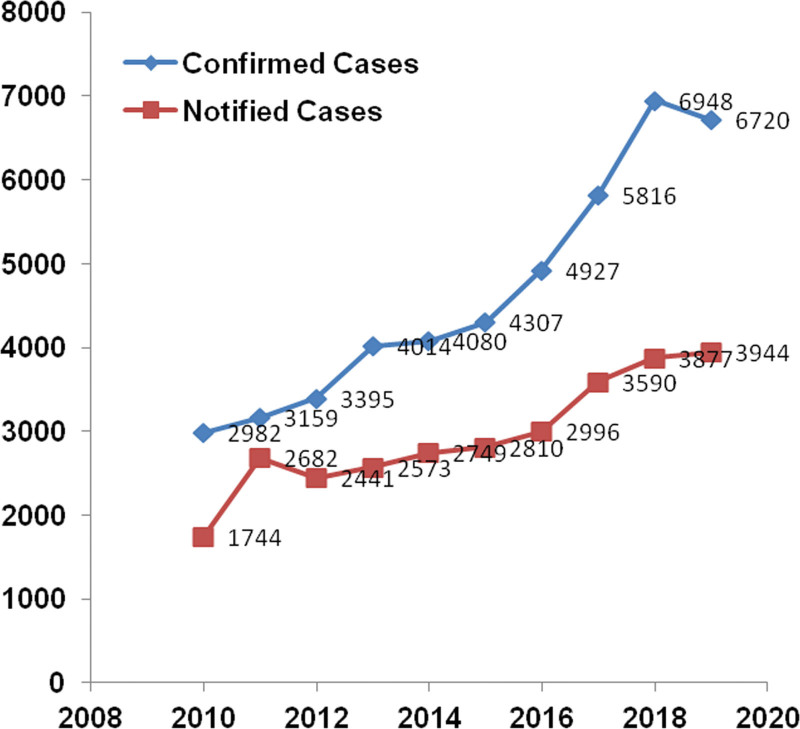
The system centralized of diagnosis and confirmation of HIV infection has led to a gap between confirmed patients and notified patients. Data between 2010 and 2019 indicate that the curve of cases notified to MINSAL is lower than the actual curve of notified cases. This finding shows that 15,835 patients were not notified despite being confirmed of having HIV. Source: MINSAL. www.minsal.cl. HIV = human immunodeficiency virus, MINSAL = Ministry of Health.

The outlook in Chile is very worrying, according to a recent report of an audit conducted in the MINSAL by the Comptroller General of the Republic; despite the large investments in therapy, Chile failed to lower the rate of new annual infections, which have been increasing in 2010 to 2019 (Fig. 1).^[3]^

Based on this report, one of the main causes of the sustained increase in new cases is the low investment in prevention policies.^[4]^

Chile has inadequately managed the HIV pandemic for several years. From 2010 to 2019, the rate of new HIV cases dramatically increased to 133% in young people aged 15 to 39 years.^[1,4,5]^

Unfortunately, this misguided policy of several years has exposed thousands of young children to the virus, thus requiring them to undergo decades of antiretroviral treatment. To end the fight against HIV infection, great global effort is required to achieve early diagnosis, universal treatment, viral suppression, retention in care, and close monitoring of patients who develop resistance to antiretroviral agents.

These 5 elements are linked because failure in 1 affects the other 4. Failure to attain these objectives jeopardizes the achievement of the 95/95/95 target by 2030, when HIV is expected to cease as a global public health threat.^[6–8]^ Chile, like many other countries, failed to meet the 90/90/90 target set for 2020. Because of this failure and the occurrence of the coronavirus disease 2019 pandemic,^[9]^ it is crucial to reinforce a stronger commitment to meet the new challenges posed by the 95/95/95 strategy. The HIV pandemic will not be resolved by measures employed by a single country; a strategy involving all countries is required. Health authorities, healthcare providers, and patients themselves must make commitments. Authorities must provide a legal framework to ensure access to early and universal diagnosis and treatment. Healthcare providers should facilitate access to patient-friendly care that ensures patient retention in the center of care and enables the timely provision of laboratory test results performed in accordance with high-quality standards. Therefore, patients should be informed of the importance of adherence to therapy and medical management. It is not enough for the governments of these countries to invest only in therapy in order to achieve viral suppression in patients. The global challenge for 2030 is the prevention of new infections.^[1,6–9]^

This study is a critical view of the main factors that have contributed to the failure to achieve the 90/90/90 target in Chile, which are responsible for the progressive increase in new HIV cases in Chile from 2010 until the present. Addressing and overcoming these barriers could help Chile end the HIV epidemic by 2030.^[1]^

## 2. Change in current legislation

In Chile, 2 laws constitute a clear barrier to diagnosis: Law 19.779 of 2001 and Decree 158 on HIV/acquired immunodeficiency syndrome (AIDS) in 2005.^[10,11]^ With regard to the HIV law, article 5 of this law establishes that the *test to detect HIV will always be confidential and voluntary, and the consent of the person concerned must be recorded in writing.* The confidentiality, voluntariness, and informed consent mentioned in this article have eventually became barriers to early diagnosis. Confidentiality has made it necessary to assign patients with disease codes to protect their identity. Years ago, this was a good strategy; nowadays, managing patients based on their disease codes is discriminatory, as it makes it clear that they are HIV carriers. One way to eliminate stigma and discrimination in the screening of HIV is to perform a universal diagnosis of the virus. Thus, people who test positive for HIV are registered in the same way as those with other pathologies, that is, with their name and identification number. Voluntariness, pre- and posttest counseling, and the signing of an informed consent form are also barriers to testing and do not favor the universal diagnosis recommended by the World Health Organization (WHO). A major advancement would be to perform opportunistic screening in hospitals and clinics in which HIV testing is included as part of medical care that requires laboratory testing. In this initiative, the person only signs a consent if he or she does not agree with the HIV test (*opt out*).^[12]^

In contrast, Decree 158/04 on compulsory notification of notifiable communicable infectious diseases is another Chilean regulation that had a negative impact on the timely diagnosis of HIV, which requires that all HIV cases diagnosed in the country must be confirmed in a single laboratory at the Institute of Public Health.^[13]^

This strategy has become a major obstacle to early diagnosis and has caused delays in timely notification of HIV status. This has had a significant impact on the poor management of HIV in Chile since only patients who have been notified can receive antiretroviral treatment. The results of the audit conducted in MINSAL in 2022 indicated that 15,835 people who were confirmed as HIV carriers by the Individual Service Plan from 2010 to 2019 were not notified. The epidemiological implications of this error were also evident.^[3]^

## 3. Strengthening universal diagnosis

Currently, the great uncertainty regarding the real number of infected people is attributed to the fact that HIV diagnosis is not widely performed in Chile, the population has little to no access to appropriate diagnostic tests, and the delivery of confirmed results is not timely. A survey conducted in more than 7500 people who underwent a rapid HIV test indicated that 25% of them would never take an HIV test due to lack of time, lack of interest, fear of the result, difficulty in accessing the test, and high cost of HIV testing.^[5]^

Thus, if a universal diagnosis is not established in Chile, the 95/95/95 goal will never be reached, as our first 95% will never be achieved, and this goal will again fail. To stop the spread of HIV infection in Chile, it is essential to significantly increase the number of people tested and implement this measure as soon as possible. The 2021 WHO guidelines on HIV testing and diagnosis recommend that all populations be tested for HIV, whether in high- or low-prevalence settings. Although voluntary testing is encouraged, WHO recommends covering both groups.

Therefore, an ambitious plan for universal diagnosis or opportunistic HIV screening is needed to determine the actual number of infected individuals in the country. The continued performance of traditional diagnosis in health centers directed only to key populations with or without symptoms or people who have risk factors, in addition to the stigma associated with the test, will not solve the problem of under-diagnosis. Only a universal diagnosis will make it possible to determine the actual number of infected people and will eliminate the stigma and discrimination associated with HIV testing in the community. Universal testing has proven its efficiency when applied to specific populations, such as pregnant women or blood donors, which significantly neutralized the risk of HIV-positive children being born or cases of posttransfusion transmission. Universal diagnosis would allow for the diagnosis of HIV among asymptomatic people and enable early treatment, thus avoiding HIV transmission in the community. Universal HIV diagnosis in Chile should be a priority in the country’s public health policies.^[14–16]^

## 4. Decentralization of HIV diagnosis

As mentioned above, Decree 158/04 established the centralization of HIV diagnosis in Chile. This centralized confirmation system is inefficient for a country of more than 4000 km long; For example, in Chile, a sample from an HIV-positive person tested in a laboratory in the Arica city must be sent to the laboratory in Santiago, more than 2000 km away to confirm the diagnosis. Then, the same sample must be returned to the laboratory in Arica to contact, notify, and link the patient to care so that he or she can access the required treatment. This centralized system has led to a gap between confirmed and notified patients. Data between 2010 and 2019 indicated that the curve of cases notified to MINSAL was lower than the actual curve of notified cases (Fig. 1). This finding shows that 15,835 patients were not notified despite being confirmed to have HIV.^[3]^

This situation is considered serious in Chile, as only cases notified to MINSAL can access treatment. This centralized system does not allow early diagnosis and linkage to treatment promoted by the United Nations Programme on HIV/AIDS. In Chile, the time it takes to diagnose and link the patient to care and therapy is far from international standards, which range from 7 to 14 days. Therefore, Chile should decentralize its diagnosis of HIV.

A good example of the benefit of decentralized HIV diagnosis has been seen in children, in which the work of Le Roux, demonstrated that the use of the decentralized point of care nucleic acid test platform for childhood HIV diagnosis was a cost-effective strategy compared to centralized nucleic acid testing, since point of care nucleic acid test achieved important benefits for the early diagnosis and timely treatment of children living with HIV.^[17]^

## 5. Decentralization of HIV healthcare

In Chile, the HIV healthcare model also focuses on the clinical follow-up of patients. This means that all laboratory tests required by HIV patients, such as viral load, cluster of differentiation 4 (CD4) T-lymphocyte count, and genotypic resistance tests, were performed in a single laboratory in Santiago. This leads to a series of practical inconveniences for referral centers because they pay the expenses for the transport of the samples, some of which are lost, arrive after hours, are poorly labeled, or arrive in a poor condition and must be rejected. This causes a delay in the delivery of results, which is perceived by patients as poor care, resulting in low retention in the treatment center, low adherence to treatment, abandonment of therapy, and desertion of medical care. For efficient management, MINSAL should provide more autonomy to regional centers in the clinical management of their patients. In other words, the HIV healthcare model should be decentralized.^[18,19]^

## 6. Implementation of self-test

In Chile, due to the number of new annual cases registered since 2010 and the coronavirus disease 2019 pandemic, which has caused an evident under-diagnosis owing to the home confinement of the population over the 2-year period, the promotion of innovative approaches to increase testing has become an urgent concern.^[9]^

Self-administered tests, also called self-assessment tests or self-tests, are performed by the patients themselves.^[20]^

Self-testing is a process in which individuals collect their own samples (oral fluid or blood) using a simple rapid HIV test and then perform the test and interpret their results at the time and place of their choice. This self-testing strategy is strongly suggested by the WHO, as it removes barriers to obtaining a diagnosis. When this type of testing is implemented in the community, traditional HIV tests performed in established laboratories significantly increase the rate of screening. Since HIV self-tests have similar levels of sensitivity and specificity to the rapid tests already used in our country, MINSAL should encourage the sale of kits used for this type of test to ensure its availability to the entire population. Self-testing is a convenient and confidential option for HIV testing as it offers people the opportunity to determine their HIV status at the time and place of their choice. It also provides complete privacy to those concerned with confidentiality. HIV self-testing is safe and accurate, and users can perform the self-test and achieve results comparable to those of tests performed by health care professionals.

To take full advantage of this diagnostic strategy, manufacturers should include a booklet with all clear indications for performing the test, along with instructions and guidelines for interpreting the results obtained. The leaflet indicates that the HIV self-test is a preliminary diagnostic guidance test and does not provide a definitive diagnosis of HIV. A reactive HIV self-test result is not equivalent to a positive diagnosis. A positive condition requires a supplemental confirmatory test, such as a nucleic acid-based assay (e.g. quantitative polymerase chain reaction). Similarly, the instructions should clearly guide the user to the next process if a nonreactive result is obtained, which can be associated with a negative result only if the user follows the window period of the test indicated by the manufacturer. Additionally, telephone numbers and other means of contact should be included. Self-testing has become an effective tool for expanding HIV testing services among people at risk of HIV infection who would not be able to undergo testing and those at ongoing risk who need frequent testing. The use of self-testing has expanded worldwide and is helping countries achieve their national and global goals.^[21]^

## 7. Creation of HIV point of care (HIV POC)

Another useful strategy that could be implemented throughout Chile is the HIV health care point (point of care human immunodeficiency virus).^[21,22]^

This modality allows diagnosis and confirmation at the same location where the patient arrives for treatment. Point of care (POC) is a combined diagnostic strategy since, in addition to serological and molecular testing; it includes CD4 T-lymphocyte count testing. POC typically uses a serological screening test that detects p24 antigen and antibodies or a molecular biology test such as a qualitative polymerase chain reaction (PCR), whose sensitivity for HIV-1 and HIV-2 diagnosis has already been validated. A positive specimen can be confirmed using a second supplemental confirmatory test, such as the Geenius method, which is based on western blot methodology that has the ability to discriminate HIV-1 from HIV-2. This test has an overall sensitivity of 99.3% and a specificity of 98%.^[21]^ Alternatively, it can be confirmed with qualitative and quantitative PCR designed and validated for HIV diagnosis.^[23]^

In this way, the patient’s HIV status can be determined in less than an hour. Using the patient’s clinical data obtained at the same point of care, the treating physician can accurately determine whether the patient is positive or negative for HIV. If the patient is confirmed to be an HIV carrier, the physician will be able to provide a prescription so that the patient can start treatment. For patients already confirmed and on antiretroviral treatment, POC is also useful as it allows the analysis of therapy failure, as it monitors patients’ adherence to treatment and can be performed on the same day as the medical visit. POC also improves clinical management of patients. The decentralized model of care improves the timely delivery of results, ensures the quality of results, and improves patient satisfaction, which, in turn, has a positive effect on adherence and retention in the clinic.^[19]^

## 8. Dual PEP/PrEP implementation

HIV preexposure prophylaxis (PrEP) involves the use of ARVs to reduce the risk of HIV infection. This strategy is effective and safe for people at a high risk of infection. There is abundant evidence supporting the use of PrEP to prevent infection.^[24–28]^ Although PrEP has been implemented in Chile, only a few centers are able to prescribe it. Therefore, authorities should extend the implementation of this strategy to all HIV centers in the country.

Although PrEP is considered effective, its effectiveness in preventing HIV transmission during emergencies, such as rape, sharp accidents, or condom breakage during sexual intercourse has not been reported. In these situations, postexposure prophylaxis (PEP) is required, which involves taking medication after possible exposure to HIV to avoid developing the infection.^[27]^

The success in decreasing new HIV cases in New York City was achieved thanks to the combined use of PrEP and PEP (*Mauro Schechter, Personal Communication*). Chile initiated the use of the PrEP protocol in 2018 in only 8 centers that would cover a total of 5000 people; to date, no more than 800 individuals have been included, and the results are evident. PrEP alone has not been successful in lowering the incidence of new infections in our country.

How effective is PEP? A previous meta-analysis included 25 relevant studies (including 408 primates). The risk of seroconversion was 89% lower among animals exposed to PEP than in those that did not receive PEP (odds ratio, 0.11 [95% confidence interval])^[28]^ Thus, PEP, if taken within 72 hours from a potential exposure, is highly effective in preventing HIV infection. This evidence supports the use of PEP by healthcare professionals who have experienced sharp injuries. Although PEP is not 100% effective in preventing HIV infection, it is an ethical duty to provide the population with this preventive alternative in the event of a risk situation, such as condom breakage during sexual intercourse, sharing needles, syringes, or other drug injection equipment, or sexual assault. Unfortunately, MINSAL Circular No. 2 of 2022 considers only PEP for victims of sexual violence. PEP should be available 24/7 in all health and emergency centers, both public and private; for this strategy to be successful, all healthcare providers should be trained, and the public should be informed in a timely manner that this prevention alternative is available prior to its launch in the country.

## 9. Genetic resistance of HIV to antiretroviral drugs

If HIV genetic resistance to antiretrovirals is not urgently addressed, the increasing incidence of resistance to therapy could jeopardize the success of efforts to scale up antiretroviral treatment and broader HIV response. According to WHO data, the rate of HIV resistance has been increasing since 2010; as treatment coverage is expanded to the entire affected population, this phenomenon could increase further. Hence, the therapy is aimed at inhibiting viral replication to allow the patient to reach an undetectable viral load. When undetectability was not achieved even after measuring 2 successive viral loads, virological failure must have occurred. Hence, it is necessary to investigate whether this response is due to either poor adherence or genetic resistance of HIV to therapy that allows it to replicate in the presence of drugs. WHO suggests maintaining close surveillance of the resistance phenomenon by implementing baseline genotyping before initiating treatment and performing more frequent genotyping.^[29]^

There are 2 types of resistance: acquired resistance, which arises when a patient is undergoing antiretroviral treatment, and transmitted resistance, which is detected in the HIV genome when the patient has not yet started therapy. In Chile, the rate of primary resistance exceeds 10%; therefore, basal HIV genotyping should be performed. It is important to identify the factors that predispose HIV patients to drug resistance. These factors include a basal CD4 T-lymphocyte count^+^ of <200 cells/µL, basal viral load of >10,00,000 copies/mL, and decreased adherence to treatment.^[30]^

## 10. Poor assessment of levels of adherence to treatment

The adherence rate is defined as the number of doses taken divided by the number of doses prescribed.^[31]^ In the case of HIV infection, adherence to treatment is considered adequate when it ranges from 90% to 95%. Below this rate, adherence to treatment cannot be guaranteed. The success of antiretroviral therapy depends on adherence to effective treatment. Different methods are used to measure treatment adherence: pill counting, self-reporting, use of a medication event monitoring system, which consists of a cap that fits on standard medicine bottles and records the time and date each time the medicine container is opened and closed, and measurement of plasma concentrations of the drug.^[32]^ In Chile, the first 2 methods are primarily used by physicians to measure their patients’ adherence to treatment. In our country, the adherence level should be measured in a more objective and reliable manner. In Canada, one of the first countries to achieve 90/90/90 goals, the method used to evaluate the degree of adherence is the measurement of drug plasma levels using high-performance liquid chromatography.

## 11. Poor sex education

The high infection rates among individuals aged 15 to 29 years show that the vast majority of young children do not use condoms as a method of protection against sexually transmitted infections. Most of them only associate condom use with avoiding pregnancy. This generation has an extremely low perception of the risk of contracting HIV infection. In addition, they consider that the availability of therapy would no longer be a problem if they contracted the virus, and only a few knew the sexual practices with the highest risk of contracting the virus.^[33,34]^

To prevent contracting sexually transmitted diseases, it is necessary to promote and strengthen sex education in the population beginning in childhood. However, this has not been the case in Chile as sex education has been undervalued as discipline at all educational levels. This shortcoming in public health policies has inspired the management and prevention of HIV infection and other sexually transmitted diseases. Several factors have contributed to the lack of sexual health education. One of these factors is the country’s social, cultural, and political characteristics. For many years, Chile has been led by the conservative economic elite with strong ties to the Church and Catholic religions. There is enough evidence to indicate that this power group has always opposed, for example, the “Days of Conversation on Affectivity and Sexuality” developed between 1994 and 2000.^[33]^

For many years, the ruling class has determined what was taught in Chile’s public and private schools. This conservative class, which was also present in political parties, had a negative influence on decisions regarding public health policies. For example, conservative sectors have always been opposed to the dissemination of condoms as a method of HIV prevention. Unfortunately, the same political sector owned the most important social communication media in the country, such as radio, television, and the press, which placed barriers to the promotion of governmental campaigns against HIV. The results of these poor public policies related to sexual education in our country are directly associated with the high number of new cases that Chile presents, mainly in the young population, thus becoming one of the leaders in the rate of new annual cases of HIV in Latin America and worldwide.

To address this issue, sex education should be provided in primary and secondary schools, as established by Law 20.418, which was enacted in 2010, having sex education mandatory. This educational work requires the MINSAL, Ministry of Education, specialized civil organizations, and family to lead children and young people.

## 12. Mental health and self-esteem

Unfortunately, Chile has a relatively high burden of mental illness,^[35]^ which shows a sharp deterioration worldwide due to the confinement and social restriction measures imposed in response to the coronavirus disease 2019 pandemic. Psychiatric illness is a risk factor for suicidal behavior and has been associated with a significant decrease in quality of life, physical health problems, drug use, and a high rate of premature mortality. For example, depression and schizophrenia increase the likelihood of premature death by suicide or physical health problems, such as cancer, cardiovascular disease, diabetes, or HIV infection, by 40% to 60%.^[36,37]^

Given this unfavorable mental health picture of children, it is easy to understand how the diagnosis of HIV can trigger a significant traumatic impact on the patient, as it involves social and long-term physical and psychological problems that can ultimately result in death. Negative emotional reactions, such as fear, guilt, shame, and uncertainty, can be observed in people newly diagnosed with HIV. An HIV diagnosis may seriously threaten a person’s self-esteem. A decrease in self-esteem due to stigmatization is common during the disease. Therefore, a combination of a high incidence of mental problems and high prevalence of HIV has a negative impact, which would lead to a higher risk of becoming infected and presenting an unfavorable clinical evolution. In Chile, not only must sex education be strengthened, but the mental health problem of the population must also be solved to break the relationship between mental pathology, drug use, and risk of HIV acquisition.

An indirect method to initially address the mental health of a community is to assess its degree of self-esteem, happiness, or depression. Self-esteem refers to an individual’s degree of regard or respect for themselves and is used to measure the values attributed to his or her own judgments and abilities. It is related to self-concept and is influenced by how the person is viewed by loved ones. Strengthening self-esteem is an effective tool for coping with daily life and avoiding harmful activities, such as drug use, which leads to increased exposure to risky situations such as HIV infection. Optimal self-esteem of the population could be a true psychosocial preventive method against HIV infection. A person with adequate self-esteem can not only avoid risky situations that expose him or her to HIV infection, but it will also serve as a tool to better cope with the disease.^[38,39]^

## 13. Migration and HIV in Chile

Between 2011 and 2015, 6,32,848 visa applications to stay in Chile were requested. Most migrants came from Peru (40%), Venezuela (17%), Bolivia (13.1%), Colombia (11%), Argentina (6.6%), and Ecuador (4.2%). The exponential growth of Venezuelan migration to Chile from 2010 to 2020 is particularly noteworthy; a total of 13,84,980 Venezuelans would have entered Chile. Unfortunately, this explosive and unplanned migration has generated strong pressure on healthcare. In the case of HIV, it is of utmost relevance since the healthcare systems of countries such as Chile that receive migrants must be strengthened so that the need for medical care and delivery of therapy can be met without negative consequences to local programs. Innovative solutions to this situation require a coordinated plan to ensure that migrants are guaranteed the right to health care. Finally, the consequences of migration to the HIV epidemic in our region have rarely been considered in relation to the implementation of HIV prevention, treatment, and care programs. This may present one of the most dramatic examples of how migration negatively affects local HIV care and control programs. Between 2010 and 2020, the Chilean government spent $686 million on antiretroviral treatment without achieving a single favorable rate in the control of new infections.^[3]^

Therefore, the uncontrolled influx of migrants seeking free therapy in our country may seriously affect HIV management in Chile.^[40–43]^

## 14. Underinvestment in HIV/AIDS research

Although Article 4 of Law 19.779 obliges the state to promote scientific research on HIV/AIDS and to promote prevention, treatment, and cure, the investment made in the country since 1984, when the first case of AIDS was detected in Chile, has been extremely low owing to the high negative impact of this pathology on the public health of the population.^[10]^

When the National Agency for Research and Development (ANID) was asked about the number of projects awarded and conducted from 1985 to 2022, ANID reported that it conducted a database search in the Applied Research and Innovation Subdirectorate using the keywords “HIV” and “AIDS,” which yielded 16 projects awarded related to the theme “HIV/AIDS” from 2000 to 2021. A total of CLP$ 7,27,835,000 was invested within the period of 21 years, which corresponds to US$ 8,42,402. Considering that these 16 projects correspond to 16 different groups that researched HIV within the 21-year period, each would have received an average of only $2507 per year for research. The first competition that addressed health issues of the Subdirección de Investigación Aplicada, formerly Scientific and Technological Development Promotion Fund, was the I National Health Research and Development Fund (FONIS) Competition 2004, which awarded a project related to “HIV/AIDS.” Prior to this date, no projects related to this topic were awarded in the FONIS competition. On the contrary, ANID reported that between 1991 and 2022, the period in which the National Fund for Scientific and Technological Development regular research competition was conducted, 18 projects were approved, unfortunately without indicating the amounts allocated. If the amounts similar to those provided by the FONIS competition are estimated, Chile would not have invested more than 1.6 to 1.7 million dollars within the 37 years (between 1985 and 2022) since the occurrence of the HIV pandemic. Therefore, the lack of investment in HIV research has also affected the successful management of this disease in our country. None of the projects funded in these 37 years were related to the development of HIV vaccines or clearance therapies.^[44]^

## 15. Implement prevention campaigns

**Figure 2.**
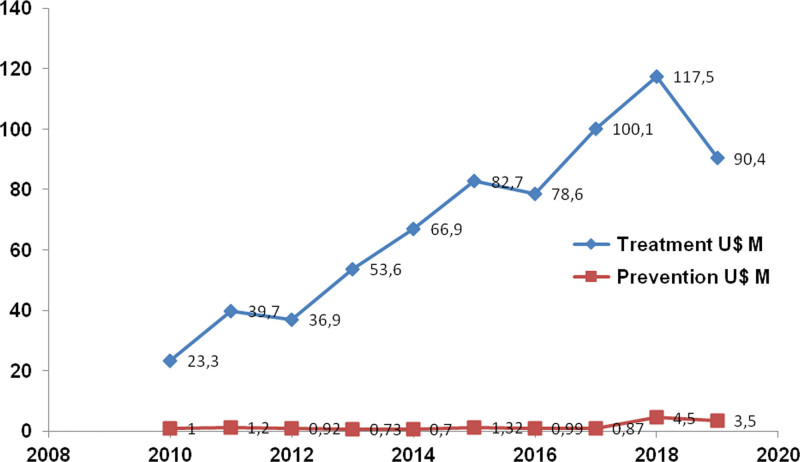
The lack of prevention policies is the main cause of the poor management of the HIV pandemic in our country. Between 2010 and 2020, the Chilean government only invested 15 million dollars in HIV prevention as opposed to the 686 million invested in treatment. In Chile with this management, a significant number of HIV patients have achieved the virological suppression, but the country failed in reducing the curve of new infections. Source: www.contraloria.cl. HIV = human immunodeficiency virus.

The lack of prevention policies is the main cause of the poor management of the HIV pandemic in our country. Between 2010 and 2020, the Chilean government invested only 15 million dollars in HIV prevention, as opposed to 686 million invested in treatment (Fig. 2). With this management, a significant number of HIV patients have achieved virological suppression in Chile, but the country has not succeeded in reducing the curve of new infections (Fig. 1).^[3,4,8]^

Government campaigns aimed at preventing HIV infection have been initiated since 1991. Between 1991 and 2009, 11 campaigns were conducted, with 1 campaign every 2 years; however, in 1998, 1999, 2000, and 2002, no campaigns were implemented.^[45]^

**Figure 3.**
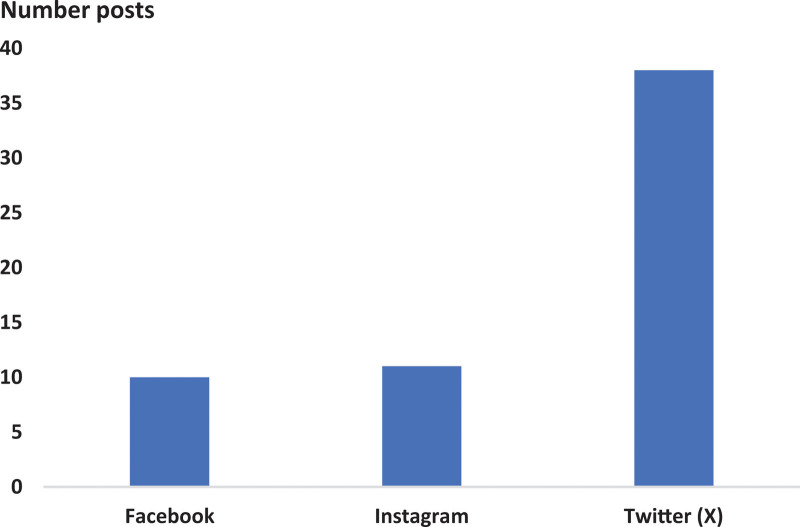
Number of publications on the social media of the Ministry of Health, Facebook, Twitter, and Instagram, related to the issue of HIV/AIDS made between 2010 and 2019. Source: www.contraloria.cl. AIDS = acquired immunodeficiency syndrome, HIV = human immunodeficiency virus.

Regarding the use of MINSAL’s social networks, it can be observed that they were very little used since the Ministry in 10 years only published 60 publications, including Facebook, Instagram, and Twitter. This equates to 1 publication every 2 months, which is an extremely low number, considering that most of the population, particularly young people, spend a lot of time connected to social networks (Fig. 3).

A report of the Chilean Congress mentions that 8 campaigns were carried out between 2010 and 2018; based on the exposure time of the campaigns, the average number of campaigns varied from 1 to 2 months and were not carried out every year.^[46]^ Between 2019 and 2022, the ministerial campaigns “It is positive to know” and “It is positive to take care,” aimed at promoting the use of condoms and HIV testing, which was only enforced for 4 months since its launch, stood out. This lack of effective and long-lasting campaigns leaves a dangerous message to the population: HIV is no longer a relevant problem to prevent.^[9]^

The new ministerial authorities have a huge task ahead of them to implement more effective HIV prevention campaigns using all the digital platforms available today; young people actively participate in social networks (RRSS) through their cell phones. The messages should be clear, simple, and direct about what actions people should follow to avoid exposure to and transmission of HIV. Messages should emphasize that years of antiretroviral treatment can cause fragility and deterioration, thereby affecting the health of people living with HIV. Through the messages sent to RRSS, a link should be sent to access free online sex education courses, inform where the nearest place is to undergo an HIV test, self-test, receive PrEP, or receive PEP.

## 16. Conclusions

Based on the above findings, we can conclude that Chile lacks successful actions taken by countries with better control of HIV/AIDS. First, the lack of sex education at all levels has generated a problem that has caused stigma and discrimination against HIV for several years. Second, preventive campaigns with direct and clear messages toward the community have remained scarce and have received little attention. Third, the implementation of centralized diagnosis does not allow timely notification of people with confirmed HIV infection, which has delayed the delivery of therapy to thousands of people. Centralized follow-up has also not helped retain patients in care centers. Better mental health and strengthening self-esteem in the population are primary tools that will help limit the risk of exposure to HIV. The country should use modern methods to objectively and reliably assess adherence by measuring drug levels in the patient’s blood. Surveillance of genetic resistance of the virus to therapy should also be improved. A balance between HIV treatment and prevention should be enforced to improve the rate of infection control. To achieve the ambitious goals proposed by the UNAIDS for 2030, Chile must accelerate the implementation of all innovative strategies proposed for this purpose, such as universal diagnosis, opportunistic screening, use of self-testing, and continuation of mass community testing.

## Acknowledgments

I would like to thank Editage (www.editage.com) for the English language translation services.

## Author contributions

**Conceptualization:** Pablo Ferrer Campos.

**Formal analysis:** Pablo Ferrer Campos.

**Methodology:** Pablo Ferrer Campos.

**Resources:** Pablo Ferrer Campos.

**Supervision:** Pablo Ferrer Campos.

**Validation:** Pablo Ferrer Campos.

**Visualization:** Pablo Ferrer Campos.

**Writing – original draft:** Pablo Ferrer Campos.

**Writing – review & editing:** Pablo Ferrer Campos.
